# [^11^C]acetate PET as a tool for diagnosis of liver steatosis

**DOI:** 10.1007/s00261-018-1558-4

**Published:** 2018-04-11

**Authors:** Marzieh Nejabat, Asha Leisser, Georgios Karanikas, Wolfgang Wadsak, Markus Mitterhauser, Marius Mayerhöfer, Christian Kienbacher, Michael Trauner, Marcus Hacker, Alexander R. Haug

**Affiliations:** 1 Division of Nuclear Medicine, Department of Biomedical Imaging and Image-Guided Therapy, Medical University of Vienna, Waehringer Guertel 18-20, 1090 Vienna, Austria; 2 Division of General and Pediatric Radiology, Department of Biomedical Imaging and Image-Guided Therapy, Medical University of Vienna, Vienna, Austria; 3 Division of Gastroenterology and Hepatology, Department of Internal Medicine III, Medical University of Vienna, Vienna, Austria

**Keywords:** NAFLD, Liver steatosis, PET, [^11^C]acetate, Liver

## Abstract

**Purpose:**

To investigate [^11^C]acetate PET-surrogate parameter of fatty acid synthase activity—as suitable tool for diagnosis and monitoring of liver steatosis.

**Methods:**

In this retrospective study, data were obtained from 83 prostatic carcinoma patients from 1/2008 to 1/2014. Mean HU was calculated from unenhanced CT of all patients from liver with liver HU less than 40 as threshold for liver steatosis. SUV_max_ of the liver and of the blood pool in thoracic aorta (as background for calculation of a liver/background ratio [SUV_l/b_]) was measured. *t* test was used with a *P* < 0.05 considered as statistically significant difference and ROC analysis was used for calculating specificity and sensitivity.

**Results:**

19/83 patients (20%) had diagnosis of hepatic steatosis according to CT. Uptake of [^11^C]acetate was significantly higher in patients with hepatic steatosis as compared to control group (SUV_max_ 7.96 ± 2.0 vs. 5.48 ± 2.3 [*P* < 0.001]). There was also a significant correlation between both SUV_max_ (*r* = − 0.52, *P* < 0.001) and SUV_l/b_ (*r* = − 0.59, *P* < 0.001) with the density (HU) of the liver. In ROC analysis for detection of liver steatosis SUV_max_ (threshold: 5.86) had a sensitivity of 94% and specificity of 69% with an AUC of 0.81. Increasing body mass index is correlated with the severity of steatosis.

**Conclusion:**

We showed for the first time that hepatic steatosis associates with increased [^11^C]acetate uptake. Also, severity of steatosis correlates with [^11^C]acetate uptake. [^11^C]acetate uptake PET seems promising for the assessment of liver steatosis.

**Electronic supplementary material:**

The online version of this article (10.1007/s00261-018-1558-4) contains supplementary material, which is available to authorized users.

Non-alcoholic fatty liver disease (NAFLD) is becoming rapidly the most frequent chronic liver disease in adult and children worldwide [1]. The incidence of disease especially in developed countries is increasing parallel to obesity and metabolic disease [2, 3] and therefore represents the hepatic manifestation of the metabolic syndrome. The US National Health and Nutrition Examination Survey (NHANES) has reported that the proportion of NAFLD among chronic liver diseases has increased from 47% to 75% between 1998 and 2008 [4]. In the US, UK and European countries NAFLD is the most common cause of chronic liver disease. NAFLD is defined by accumulation of lipid in more than 5–10% of liver weight which is not caused by excessive consumption of alcohol [5]. About 20% of patients with NAFLD can progress to non-alcoholic steato-hepatitis (NASH) which is characterized by the presence of inflammation and other signs of progressive liver injury in addition to steatosis. In laboratory chemistry, elevations of liver enzymes (AST, ALT and probably G-GT) may be observed, but poorly reflects disease activity and may even be within the normal range. The disease spectrum can further progress to liver fibrosis and liver cirrhosis [3, 6]. Alarmingly, NASH has become already the leading cause of hepatocellular carcinoma (HCC) in some European countries and NASH-associated HCC occurs in an increasing rate in pre-cirrhotic stages of NASH, posing additional challenges for screening strategies [7, 8].

The standard diagnosis of NASH is made by liver biopsy and histopathological staging [9, 10]. Currently different imaging methods are used for evaluation and quantification of NAFLD, as biopsy is invasive with risk of complications, semi-quantitative and prone to sample variability. Fibroscan™ as a method for measuring liver stiffness and Fibroscan™ CAP^®^ for the quantification of liver steatosis are becoming widely used. However, these measures do not reflect intrahepatic metabolic alterations [11]. Therefore, a non-invasive diagnostic tool for monitoring the extent of lipid accumulation and for diagnosis of NASH is urgently needed. Ultrasonography as the most accessible tool for evaluating NAFLD is limited by operator dependency, lack of quantitative information as well as not sufficient sensitivity and specificity [12–14].

Computer-tomography (CT) can quantify the density of liver tissue, which is reduced by lipid accumulation. However, CT seems also to be not sensitive enough for robust diagnosis of mild steatosis [14]. It has been reported that CT has about 97% sensitivity and 76% specificity for fat infiltration more than 33% (that would be in the range of moderate). Magnetic resonance imaging (MRI) has been recently used for diagnosis of NAFLD. In some studies it has been shown that it correlates with histology data but sequences and values are vendor dependent, so its use is hampered for follow-up or diagnosis of disease severity [15–17]. Relative liver enhancement of gadoxetic acid may also distinguish patients with NASH among NAFLD individuals with a good diagnostic accuracy as demonstrated in a Viennese cohort of NAFLD patients [18].

Positron emission tomography (PET) imaging with and without CT has emerged as a diagnostic tool for liver disease, in particular for hepatic lesions. Only few studies used PET imaging for evaluation and quantification of liver fat density and its correlation with obesity and/or coronary disease, all of them focusing on glucose metabolism measured with 2-[^18^F]fluoro-2-deoxy-d-glucose ([^18^F]FDG). However, results are contradictory and not convincing so far [19, 20].

Recently, several studies have highlighted the role of acetate as one of the most central and dynamic metabolites in intermediary lipid metabolism [21]. Acetate or acetic acid in human cells is converted into acetyl-Co A and, therefore, is involved in synthesis of cholesterol and fatty acids and plays a fundamental role in cell growth and proliferation processes [21]. A close correlation between [^11^C]acetate uptake and fatty acid synthesis has already been shown [22]. Consequently, it has been used to assess fatty acid production in cancers and heart tissue [23–26]. Despite the drawback of limited availability, [^11^C]acetate can be considered as promising tool in many diseases connected to lipid accumulation. Therefore, we hypothesize that [^11^C]acetate uptake is significantly increased in steatosis as compared to normal liver parenchyma.

## Materials and methods

### Subjects

The institutional review board of the Medical University of Vienna has approved the study. The study retrospectively reviewed 126 consecutive patients with known prostate carcinoma who have had [^11^C]acetate PET/CT scanning at the Medical University of Vienna between January 2008 and January 2014. Only patients with present non-enhanced CT were enrolled in this study. Additionally, conventional diagnostic workup including a thorough history, body mass index (BMI) and laboratory data (including ALT, AST and Gamma GT) were selected and assessed retrospectively. In most patients alcohol abuse could be ruled out. However, in some cases no information about alcohol consumption was available. The indication of PET–CT for all included patients was biochemical relapse of prostate cancer and patients who received chemotherapies at the time of PET/CT, known liver disease (e.g., viral or auto-immune hepatitis) and also patients with liver metastasis or a significant tumor burden with [^11^C]acetate uptake were excluded from the study.

### [^11^C]acetate PET/CT

[^11^C]acetate was prepared according to a well-established method [27]. All PET/CT scans were obtained using combined PET/CT scanner (Siemens Biograph TruePoint 64). According to the routine protocol all patients were asked to fast for at least 6 h before the examination and then received an intra-venous injection of [^11^C]acetate of 8 MBq/kg of body weight. After 20 min patients were scanned from thorax and abdomen. PET images were reconstructed using the TruX algorithm, with four iterations per 21 subsets, a 5-mm-slice thickness and a 168 × 168 matrix. Helical CT acquisitions were performed with 4 D care dose protocol and the following parameters: a tube current of 230 effective mAs, a tube voltage of 120 kVp, a collimation of 24 × 1.2 mm, a pitch of 0.813, and a scanning time of 0.5 s per rotation. For review, the CT images were reconstructed with a section thickness of 5 mm in 3-mm increments.

PET, non-enhanced CT and fused PET/CT images were generated and reviewed on the computer by a specialized physician, and co-registered images were displayed on special workstation system using Hybrid Viewer (HERMES Medical solutions, Stockholm, Sweden).

### Data analysis

**Fig. 1:**
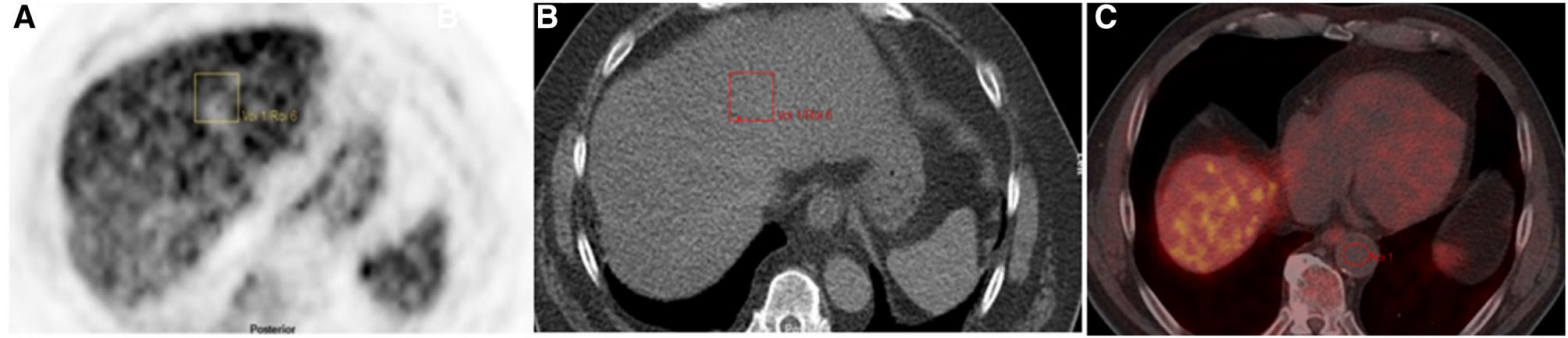
Fatty liver has been defined as mean liver attenuation measured in Hounsfield Units (HU) < 40. [^11^C]acetate uptake (**A**) and CT HU (**B**) values of the liver were measured by drawing three region of interest (ROI) with a diameter of at least 3 cm within the liver. Three ROIs into the blood pool within the thoracic aorta (**C**) to measure the SUVmean as background activity for standardization of liver uptake (calculation of liver/background SUV ratio)

PET uptake values from liver, spleen as well as blood pool of thoracic aorta and CT HU of the liver were measured by drawing three region of interest with a diameter of at least 3 cm in the center of the organ for liver and 1.5 cm for the blood pool in the thoracic aorta. Continuous data (such as BMI, SUV_max_, HU) are reported with medians, range and standard deviations while categorical ones are mentioned with counts and percentage. As defined in previous studies fatty liver has been defined as mean liver attenuation measured in HU less than 40 [28–30]. Therefore, the steatosis group was defined as study subjects with HU < 40 and the rest have been considered as control group. The maximum standard uptake value (SUV_max_) of the thoracic aorta has been considered as reference value for calculation of liver/background SUV ratio (SUV_l/b_). It has been measured by adding 3 manually drawn ROI to a VOI (Fig. 1).

Correlation between the steatosis patients according HU definition, SUV_max_ and SUVl/b was performed using Pearson correlation test and a *P* value < 0.05 was considered statistically significant. For comparison of different values of the patient group and control group, we used a *t* test for independent samples in combination with a Levene test for equality of variances. A *P* value < 0.05 was considered as statistically significant.

Sensitivity and specificity of [^11^C]acetate PET as a diagnostic tool for liver steatosis has been calculated using ROC analysis (receiver operating characteristic).

## Results

According to the inclusion criteria 43/126 patients were excluded from the study due to: liver lesions, known hepatitis B and/or C, incomplete laboratory data, lack of unenhanced CT, and additional disease or therapies that may affect liver parenchymal enhancement. The study population included 83 patients (mean age 68.9, range 48–94). 19/83 patients were defined as steatosis patients and 64 patients as controls. This data indicates a prevalence of steatosis in about 20% in our study population.

We observed a statistically significant difference in BMI between steatosis and control group, consistent with NAFLD as the most likely etiology: mean BMI measure from steatosis group was 32.3 ± 1, whereas in control group was 26.8 ± 0.5.

**Fig. 2:**
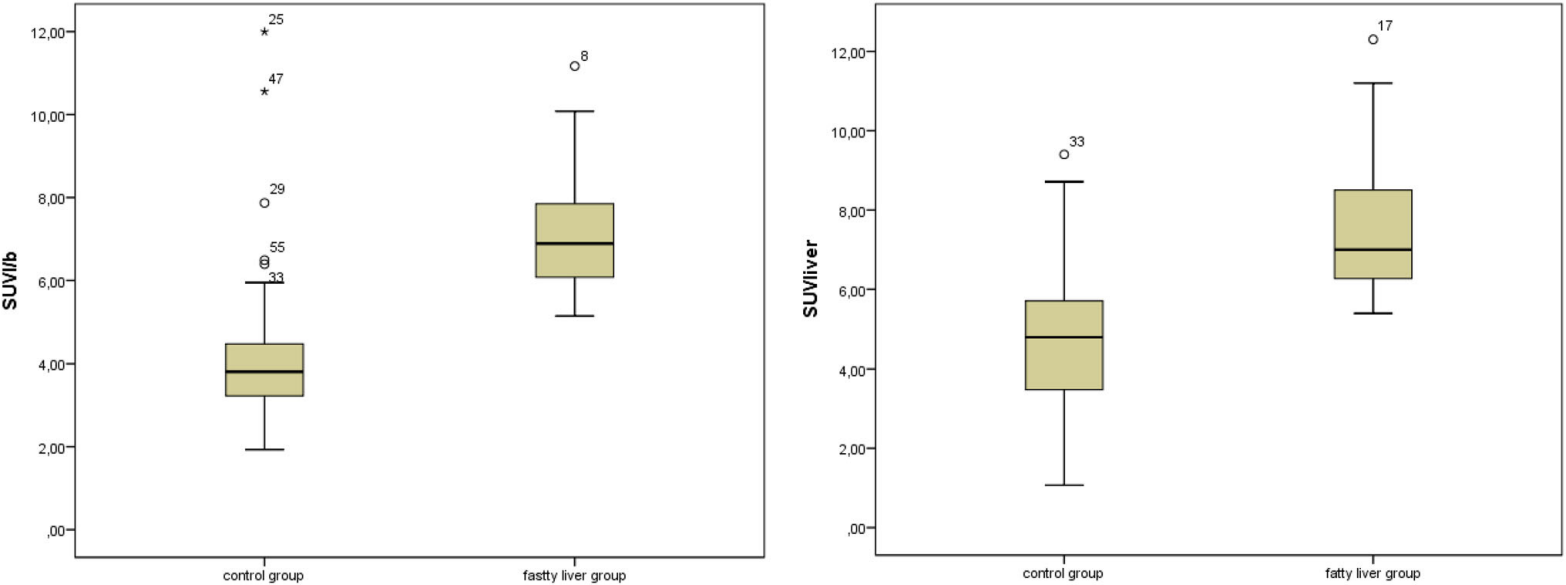
Graph illustrates [^11^C]acetate uptake of the liver measured in SUV_max_ (**A**) and SUV_l/b_ (**B**). Graph indicates significant difference between fatty liver group (1) vs. control group (0) (**A** *P* < 0.001 and **B** *P* < 0.001)

**Fig. 3:**
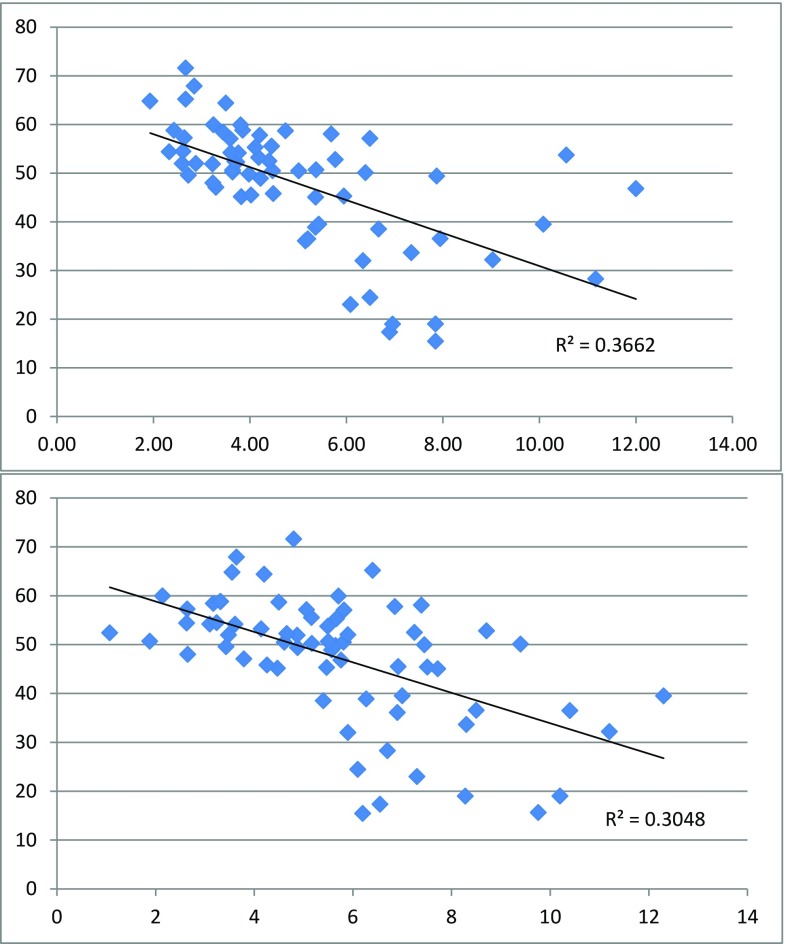
Correlation curve of liver Hounsfield (HU) and standard uptake value (SUV_max_) of liver (**A**) and SUV_l/b_ ratio (**B**) in study population. Graph indicates significant association between these parameters in SUV_max_ alone and SUV_l/b_ (respective *P* value : < 0.001)

The mean SUV_max_ of the liver in the control group (5.48 ± 2.3) was significantly lower than the SUV_max_ of the steatosis group (7.96 ± 2.0; *P* < 0.001; Fig. 2). The mean SUV_l/b_ in the steatosis group was significantly higher as compared to the control group (7.17 ± 1.7 vs. 4.78 ± 2.2; *P* < 0.001). Increasing SUV_max_ of the liver had a statistically significant negative correlation with decreasing mean HU (*r* = − 0.52; *P* < 0.001). The SUV_l/b_ and the HU liver showed a significant correlation, too (*r* = − 0.59; *P* < 0.001). This relationship is shown in Fig. 3.

Liver enzymes in fatty liver group were higher than in controls, but with the exception of AST the difference was not statistically significant (Table 1). According to available follow-up data from 15/19 patients (4 patients without follow-up data) of the control group with high [^11^C]acetate uptake but no signs of steatosis on CT, about 47% (9/19 patients) developed elevated liver enzymes and/or report of liver steatosis in abdominal CT/ultrasonography during follow-up.

**Table 1:** Summary of all demographic, laboratory and quantitative data of all study subjects generally and according disease/control group identifications

Value	All patients	Control group	Steatosis group	*P* value
Mean HU liver	47.3	53.7	29.2	< 0.001
Mean SUV_max_ liver	5.7	4.9	8.0	< 0.001
Mean SUV_l/b_	5.0	4.3	7.2	< 0.001
Mean BMI	28.0	25.8	32.3	0.001
Mean ALT	32.0	29.4	38.3	0.2
Mean AST	33.9	31.3	40.7	0.3
Mean G-GT	97.1	106.7	73.9	0.6
Mean age	68.9	69.2	70.5	0.6

**Fig. 4:**
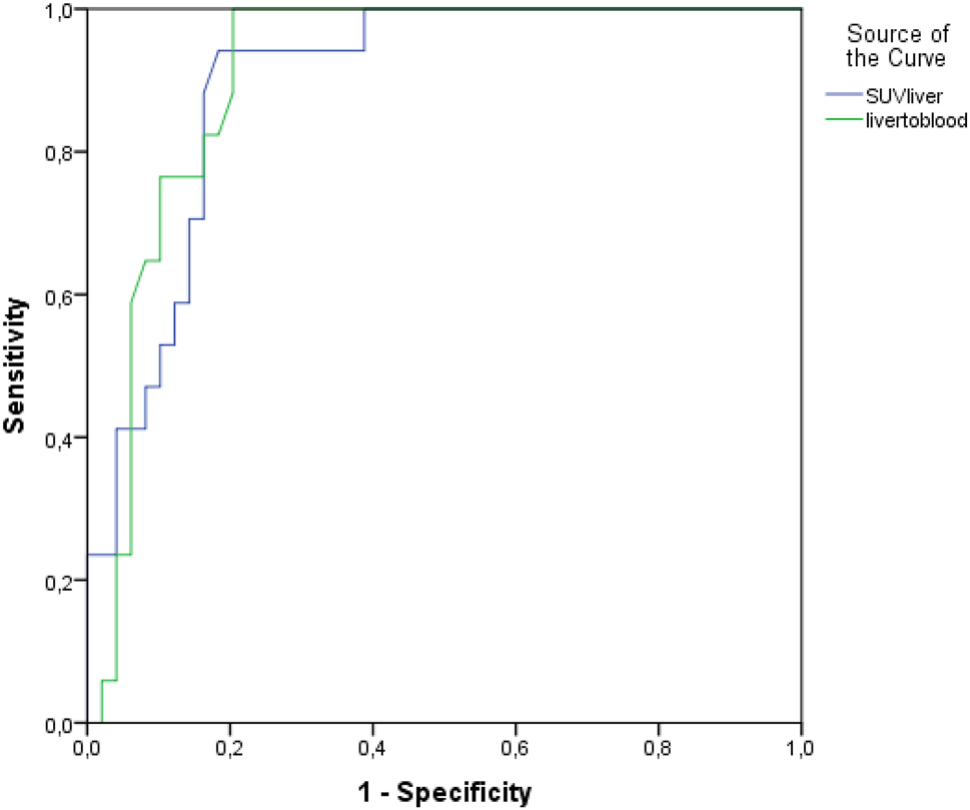
ROC curves for detecting sensitivity and specificity of [^11^C]acetate PET for SUV_max_ alone (blue line; AUC = 0.78) and SUV_l/b_ (green line AUC = 0.80)

The sensitivity of [^11^C]acetate PET/CT for diagnosis of steatosis patients was analyzed using ROC analysis. The value of steatosis diagnosis by using SUV_max_ alone, as well as using SUV_l/b_ is shown in Fig. 4. SUV_max_ (AUC 0.81) had a sensitivity of 94% and specificity of 69%, whereas SUV_l/b_ (AUC 0.84) had a sensitivity of 77% and specificity of 82%. The biochemical as well as clinical characteristics and imaging information of study participants categorized by presence or absence of steatosis are presented in Table 1.

For further analysis, we excluded all patients with a borderline HU value of the liver between 40 and 45 (*n* = 13). We aimed for a more robust differentiation of patients and tried to exclude patients with borderline fatty involvement of the liver. In the remaining patients, SUV_max_ (7.96 vs. 4.89; *P* < 0.001) and SUV_l/b_ (7.17 vs. 4.27; *P* < 0.001) of the steatosis group was significantly higher as compared to the control group. HU of the liver and SUV_max_ (*R* = − 0.60; *P* < 0.001) and SUV_l/b_ (*R* = − 0.57; *P* < 0.001) correlated significantly. Using ROC analysis SUV_max_ had a sensitivity of 94% with a specificity of 82% (AUC = 0.89; threshold SUV_max_ 5.86). SUV_l/b_ had a sensitivity of 100% and a specificity of 80% (AUC = 0.91, threshold SUV_max_ 5.08) (Fig. 4).

## Discussion

According to recent guidelines published by the American Gastroenterological Association in patients with the incidental findings of fatty liver (mostly in ultrasound), liver biopsy is not recommended in the absence of risk factors such as diabetes mellitus, hyperlipidemia, hypertension and/or central obesity. These patients should be followed by an imaging method and by monitoring of biochemical data [31]. Several imaging methods are available for diagnosis and follow-up of NAFLD, all of them with specific drawbacks. Ultrasound is machine and operator dependent and especially in patients with central obesity and thick adipose tissue, it cannot be used properly [32]. CT has been shown to have less accuracy in detecting mild steatosis in comparison to more advanced steatosis [28]. MRI is prone to artifacts and has difficulties in providing reliable vendor-independent quantitative data. As mentioned before, despite its increasing use, Fibroscan does not reflect the metabolic situation [11]. Further on, results of Fibroscan show a wide variation in sensitivity ranging from 77% to 100% and specificity ranging from 78% to 98% [33].

We proposed [^11^C]acetate PET as a marker for hepatic steatosis, because of its involvement in free fatty acid metabolism cascade, which has a reasonable relevance and, therefore, can be used especially in high-risk patients for diagnosis, risk stratification and follow-up. About 25% of fatty acids for hepatic triglyceride accumulating in NAFLD result from increased de novo lipogenesis which is driven by insulin and dietary factors including fructose [34]. In our study, not surprisingly, we have had a high incidence of steatosis as an incidental finding in the study population (20%) [35] that underlines the necessity of an optimum imaging method for primary diagnosis as well as follow-up evaluation of a population at risk. With these results, for the first time we could demonstrate the utility of [^11^C]acetate PET for detecting fatty accumulation in the liver. In our study, liver SUV_max_ and SUV_l/b_ had a high accuracy in detecting fatty infiltration with liver HU as gold standard (sensitivity of 94% and specificity of 69% for liver SUV_max_ and sensitivity of 77%, and specificity of 82% for SUV_l/b_). Furthermore, we demonstrated that the severity of fatty infiltration according CT findings was correlated with increasing [^11^C]acetate uptake. As expected the fatty liver group had significantly higher BMI in comparison to control group and a trend of higher liver enzymes was observed in fatty liver group that both correlated with findings in relationship between fatty liver and obesity/inflammation. When excluding patients with a borderline density of the liver (HU between 40 and 45) for further analysis [^11^C]acetate had an even higher AUC for diagnosis of fatty liver disease. Excluding patients with a borderline liver density in CT was done for this additional analysis as a value of 40 HU for discriminating patients with fatty liver disease is quite arbitrary and the transition to fatty liver disease is smooth. We suggested that patients with a liver HU of 40–45 might present with increased fat in the liver without fulfilling the CT-based criteria and excluding these patients might allow for better discrimination of patients with and without fatty liver disease.

During recent years, few studies were published analyzing [^18^F]FDG uptake in the liver for detecting fatty infiltration. The results were controversial: in a retrospective study Jonathan et al. [19] could not find any significant difference in [^18^F]FDG uptake between a fatty liver group and a control group, whereas Qazi et al. [36] described a significantly higher [^18^F]FDG uptake in the fatty liver group compared with healthy controls. From the metabolic point of view, increasing glucose metabolism in patients with fatty infiltration—especially with mild or no inflammation—seems not to be likely. Also considering the high percentage of patients with NAFLD suffering from diabetes mellitus or metabolic syndrome a basic disturbance in glucose uptake in whole body organs as well as the liver must be assumed. Another study has proven this glucose uptake disturbance theory by demonstrating an inverse association between liver fat content and [^18^F]FDG uptake in type 2 diabetic patients [37]. In this study, [^18^F]FDG was injected 90 min after insulin stimulation in diabetic patients. This could be relevant for type 2 diabetic patients and patients with metabolic syndrome, although the correlation of insulin resistance with the fat content of the liver is questionable.

Due to the lack of biopsy-proved studies the accuracy of PET, CT or MRI in detecting mild-to-moderate grades of NAFLD is not clear. Although there are studies showing that assessment of liver fat by CT attenuation is unreliable and is insensitive for detecting mild steatosis, the reported sensitivity and specificity of unenhanced CT for detecting moderate-to-severe steatosis (> 30% on histology) is about 73% and 95%, respectively [38]. Therefore, the gold standard for diagnosis of NAFLD has limitations in its accuracy itself. Indeed, we noted some patients with high [^11^C]acetate uptake in the control group. Interestingly, 47% of these patients with increased [^11^C]acetate uptake developed NAFLD later on. Therefore, we hypothesize that an increased [^11^C]acetate uptake might precede development of morphologically visible liver steatosis. In addition, our study population were oncologic patients, consisting of males only with somehow high age range and a higher likelihood of presence of chronic diseases; all these factors might affect normal parenchyma uptake in patients. For establishing a normal range of liver [^11^C]acetate uptake or a reliable cut-off value for NAFLD, a healthy control group would be needed. Another clear limitation of the study was the lack of histopathologic data as the detection of fatty liver was retrospectively done and can be considered as an incidental finding.

Finally, our results apply to a cross section of patients in a single period of time. Therefore, we could not demonstrate the utility of [^11^C]acetate PET for reliable non-invasive monitoring of NAFLD; however, the increasing uptake with increasing fat content is an indicator for its potential.

As mentioned before, [^11^C]acetate PET provides a functioning image from the fatty acid content of the liver cells, which might allow for future non-invasive analysis of interventions against NAFLD. Some studies have shown that dynamic PET imaging possibly including kinetic modeling may give us more accurate information, for example in diagnosis of focal, multifocal or geographic liver steatosis. In a recent study, [^11^C]acetate was used for detection of FNH and primary HCC and the accuracy of diagnosis was significantly increased when dynamic imaging protocols were used [39].

In conclusion, the presented data demonstrated a significant association between liver attenuation—and therefore liver fat content—and [^11^C]acetate uptake for the first time. We can conclude that [^11^C]acetate PET can be considered as a promising and reasonable imaging biomarker for early diagnosis and follow-up of patients with fatty liver. This should be the basis for further studies to evaluate the accuracy of [^11^C]acetate PET as a diagnostic tool for detection of different grades of fatty liver disease (mild, moderate and severe), for evaluation of follow-up situations, prognostic risk stratification, and monitoring therapeutic interventions.

## Electronic supplementary material

Below is the link to the electronic supplementary material.

Supplemental Fig. ROC analysis excluding all patients with a liver HU from 40 to 45. In this population sensitivity, specificity and AUC are clearly higher than in the total population (SUVmax: sensitivity 94%, specificity 82%, AUC = 0.89; SUVl/b: sensitivity 100%, specificity 76%, AUC = 0.88). Supplementary material 1 (JPEG 385 kb)
