# Supplementary figures and images for: [11C]acetate PET as a tool for diagnosis of liver steatosis
Source: Abdom Radiol (NY). 2018 Apr 11;43(11):2963–9. doi: 10.1007/s00261-018-1558-4 (PMC6208816; doi:10.1007/s00261-018-1558-4)

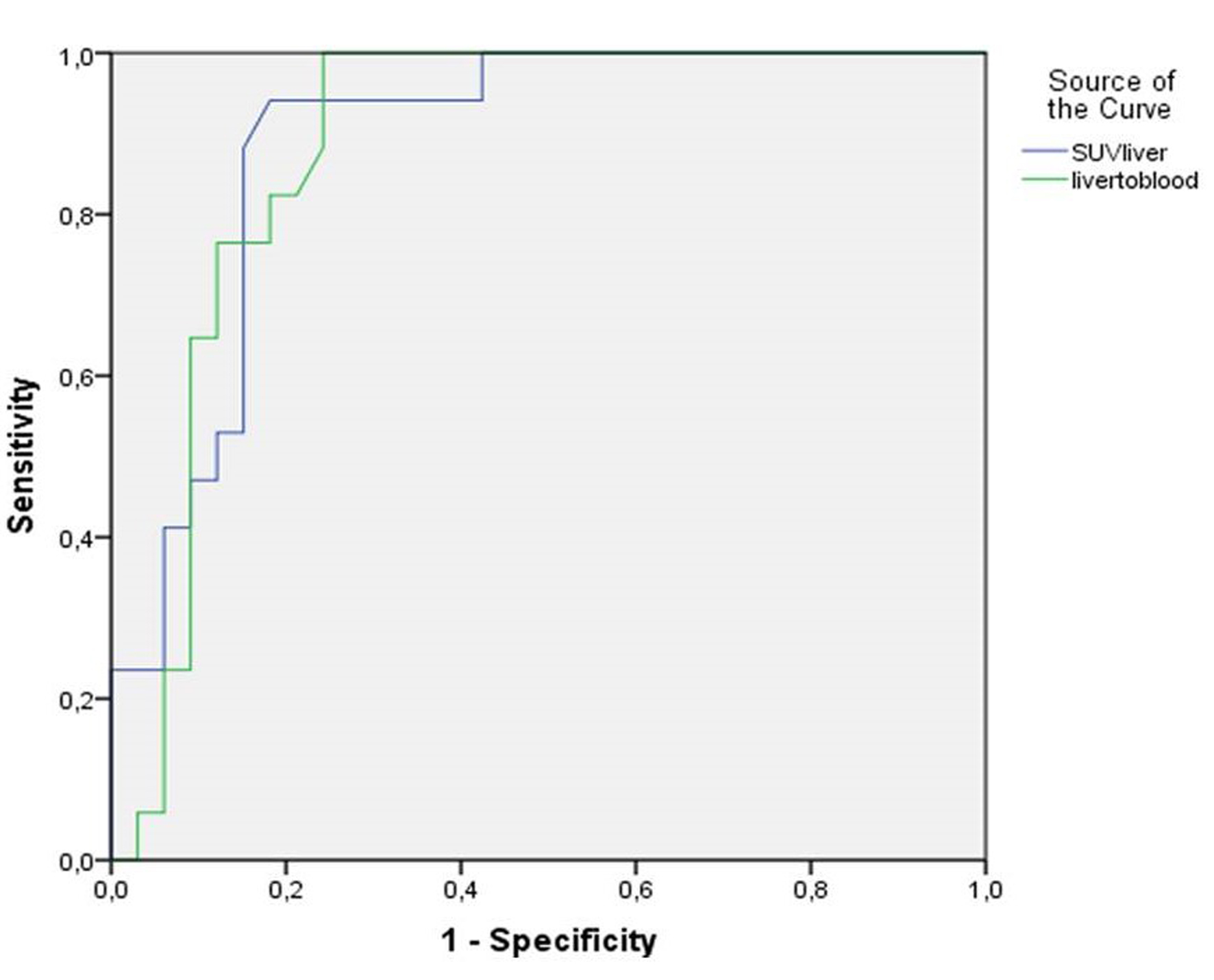

Supplement: Supplementary file 1 — ROC analysis excluding all patients with a liver HU from 40 to 45. In this population sensitivity, specificity and AUC are clearly higher than in the total population (SUVmax: sensitivity 94%, specificity 82%, AUC = 0.89; SUVl/b: sensitivity 100%, specificity 76%, AUC = 0.88). Supplementary material 1 (JPEG 385 kb) [file 261_2018_1558_MOESM1_ESM.jpg]
